# Training requirements and associated factors for nursing assistants in Jiaxing City, China: a cross-sectional study in healthcare settings

**DOI:** 10.3389/fmed.2026.1710478

**Published:** 2026-02-12

**Authors:** Feng-Yan Pu, Li-Ping Yu, Hui-Ping Shan

**Affiliations:** 1 Department of Nursing, The Second Affiliated Hospital of Jiaxing University, Jiaxing, Zhejiang, China; 2 Department of Radiology, The Second Affiliated Hospital of Jiaxing University, Jiaxing, Zhejiang, China

**Keywords:** influencing factors, nursing assistant, training demand, training status, workforce development

## Abstract

**Background:**

The aim of this study is to systematically evaluate the training requirements of nursing assistants in clinical settings and to identify influencing factors, with the objective of developing a training requirements model to inform the design of evidence-based training programs.

**Methods:**

A cross-sectional design was implemented using stratified sampling across 48 healthcare institutions in Jiaxing City. A total of 1,600 questionnaires were distributed to nursing assistants, yielding 1,470 valid responses (valid response rate: 91.9%). Descriptive statistics, chi-square (χ^2^) tests, and multivariate logistic regression were conducted using SPSS version 22.0 to assess training coverage, content, format, and influencing factors.

**Results:**

High training coverage was observed for both pre-employment (92.6%) and on-the-job (87.0%) programs; however, the reported training durations were relatively brief. Instruction was primarily delivered via in-hospital lectures and traditional mentorship models. Key barriers to training participation included scheduling conflicts, technological limitations, and inadequate practical content. A substantial proportion (78.4%) of respondents indicated interest in retraining. Multivariate analysis demonstrated that nursing assistants with longer work experience or those employed through labor dispatch or hired directly by families exhibited lower training requirements. In contrast, higher training requirements were identified among those working under one-on-one or one-to-many care models, and among those who had previously received on-the-job training (all *p* < 0.05). Preferred training formats included short-term (1–3 day) sessions held at high frequency (monthly), facilitated by experienced nursing assistants and subject-matter experts. Hospitals were the most preferred venues, with on-site mentorship identified as the favored instructional method. The most desired training topics among participants were qualities and requirements of nursing assistants (67.3%), particularly communication techniques (59.2%), as well as content related to practical skills.

**Conclusion:**

Despite high overall training coverage, several limitations were identified, including brief training durations, limited instructional diversity, and underutilization of digital platforms. Enhancing the practical relevance of training content, incorporating blended learning approaches that combine online and in-person methods, and optimizing both training frequency and duration are recommended to improve engagement and training outcomes among nursing assistants.

## 1 Background

China, now navigating the world’s most rapid demographic aging, confronts healthcare challenges of an unprecedented scale. Projections suggest that by 2050, over 400 million citizens will be aged 65 or older (1, 2). This profound demographic transition, occurring against a backdrop of high chronic disease prevalence and a growing number of empty-nest households, is intensifying the societal demand for long-term care. The traditional reliance on family-provided care is increasingly strained by sustained low fertility rates, exposing the vulnerabilities of an informal system where caregivers often lack professional skills and face their own health challenges (3).

This growing care deficit intersects with a critical constraint within the formal health sector: a global shortage of registered nurses (4, 5). Inadequate nurse staffing not only fuels occupational burnout and turnover but also compromises the consistent delivery of safe, high-quality care (6). In response, health institutions across China have come to depend significantly on nursing assistants, often termed “healthcare caregivers,” to perform essential, non-specialized tasks under professional supervision (7). Unlike licensed nurses, this workforce enters the system without standardized formal education, creating a foundational challenge for quality assurance (8).

The current profile of nursing assistants in China, typically older, with limited formal education (72.71% completed junior high school or less), and exhibiting high turnover, further complicates the development of a skilled caregiving cohort (9). The central dilemma lies in this paradox: an escalating need for high-quality care is being met by a workforce for which effective, standardized training is not yet fully realized.

Existing literature adequately outlines these structural and educational challenges in broad terms. However, a critical gap persists: a lack of granular, context-specific evidence detailing how these general challenges translate into concrete, actionable training needs for nursing assistants at the local level. Studies that move beyond generic descriptions to identify the specific factors influencing training requirements within distinct socio-economic contexts, such as Jiaxing, a rapidly aging yet economically vibrant city in the Yangtze River Delta, are notably absent. This gap limits the development of targeted training frameworks.

To address this, our cross-sectional study investigates the existing training landscape for nursing assistants in Jiaxing and identifies key factors shaping their training requirements. The findings are intended to inform the creation of a more systematic, standardized, and tiered training approach, which is becoming an urgent necessity for building a resilient healthcare workforce capable of meeting the demands of an aging China.

## 2 Materials and methods

### 2.1 Design

A cross-sectional study design was employed.

### 2.2 Setting

The study was conducted in Jiaxing City, located in the core area of the Yangtze River Delta, China. Stratified sampling was conducted across seven sub-centers under the Jiaxing Nursing Quality Control Center, involving 48 healthcare institutions. These included tertiary Grade A and B hospitals, secondary Grade A and B hospitals, and long-term care facilities, ensuring adequate representation of institutional diversity. Before data collection, a preparatory meeting was held with the directors of all seven sub-centers. To ensure consistency and data quality, each sub-center director recruited 3–4 research assistants who underwent standardized training before participating in the study. A total of 28 trained research assistants were responsible for distributing questionnaires and guiding nursing assistants in completing them. Given that many nursing assistants were older and had lower educational levels, research assistants administered the questionnaires orally when necessary and recorded responses accordingly. Prior to data collection, research assistants received formal training, and all participants were provided with a brief explanation of the study’s purpose and significance.

### 2.3 Participants and samples

Participants were from 48 different health care institutions in Jiaxing. Stratified sampling was utilized to recruit nursing assistants employed at these institutions. Inclusion criteria were: ➀ aged 18 years or older; ➁ possessing adequate communication skills; and ➂ voluntary participation with informed consent. The required sample size was estimated based on statistical recommendations indicating a sample size of 10–20 times the number of variables (10). Considering a 10% expected attrition rate and a questionnaire comprising 60 items, the minimum required sample size was calculated to be 667 participants.

### 2.4 Measurement

Two instruments developed by Wang were used to collect data (11): the In-Service Training Status Questionnaire for Nursing Assistants and the In-Service Training Needs Questionnaire for Nursing Assistants (Supplementary material). The content validity index (CVI), including both item-level and scale-level indices, was evaluated by a panel of 10 experts specializing in nursing assistant training (2 directors of nursing departments, 6 clinical head nurses, and 2 faculty members from nursing colleges). The In-Service Training Status Questionnaire demonstrated a CVI of 0.904, test-retest reliability of 0.883, and a Cronbach’s α coefficient of 0.869. The In-Service Training Needs Questionnaire reported a CVI of 0.910, test-retest reliability of 0.893, and a Cronbach’s α coefficient of 0.879.

The questionnaire used in this study consisted of the following sections: (1) General information of the participants: including gender, age, educational background, and years of professional experience. (2) Current training status: whether the participant had received pre-employment training, duration of the pre-employment training, whether in-service training had been received, duration of in-service training, as well as the trainer’s background and the training location. Participants were also asked to indicate the difficulties encountered during pre-employment or in-service training (multiple choices allowed). (3) Training needs: whether the participant had a need for retraining, preferred training duration, preferred training methods, trainer background, and desired training content (multiple choices allowed).

### 2.5 Procedures

The survey was administered electronically using the “Wenjuanxing” (Questionnaire Star) platform. A total of 28 research assistants were recruited through the seven sub-centers. To support data integrity, research assistants visited participating institutions to assist with questionnaire administration. Before formal data collection, all research assistants received training on standardized procedures. Given the older age and relatively low educational levels of many nursing assistants, oral explanations and assistance with questionnaire completion were provided when needed. All respondents received a brief explanation outlining the study’s purpose and significance. Informed consent was obtained through a digital prompt requiring participants to select either “Yes” or “No.” Those who selected “No” were automatically excluded from participation. Participants were informed of their right to withdraw at any time. Once consent was confirmed, the questionnaire could be completed using mobile phones, computers, or other electronic devices. To prevent duplicate responses, each questionnaire could be submitted only once. Incomplete questionnaires were excluded from the final analysis. The data were stored and verified by two principal investigators to ensure accuracy and confidentiality.

### 2.6 Analysis

Data were analyzed using SPSS version 22.0 (IBM Corp., Armonk, NY, United States). Categorical variables were summarized as frequencies and percentages. Differences in retraining requirements (yes/no) across demographic and occupational characteristics were examined using the chi-squared (χ^2^) test. Variables with *p* < 0.05 in univariate analyses were entered into a multivariate logistic regression model (enter method) to identify independent factors associated with retraining requirements. The Omnibus Tests of Model Coefficients was used to assess the overall significance of the model coefficients. In this study, *p* = 0.001, indicating that at least one of the variables included in the model has an odds ratio with statistical significance; in other words, the model as a whole is meaningful. The Hosmer and Lemeshow Test was used to evaluate the goodness-of-fit of the model. Here, *p* = 0.544, suggesting that the information in the current dataset has been adequately captured by the model and that the model demonstrates a good fit. All statistical tests were two-tailed, with a significance level set at *p* < 0.05.

## 3 Results

### 3.1 Current training status of nursing assistants in Jiaxing

Of the 1,600 questionnaires distributed, 1,470 were valid (response rate: 91.9%). As shown in Table 1, coverage of pre-employment (92.6%) and on-the-job training (87.0%) was high. However, training was predominantly short in duration: 35.3% of participants received only 1–3 days of training, and 31.0% received 4–7 days. Hospital-based lectures (63.4%) and traditional mentorship (66.5%) were the most common training formats. Key barriers included scheduling conflicts (38.0%), limited smartphone use (31.7%), and insufficient practical content (9.7%). Despite these constraints, most participants (78.4%) expressed willingness to attend retraining. Among those unwilling, age-related limitations (40.7%) and lack of time (34.1%) were the primary reasons.

**TABLE 1:** Current training coverage, format, duration, and barriers among nursing assistants in Jiaxing, China (*n* = 1,470).

Item	Content	Number of cases	(%)
Pre-employment training	Yes	1,361	92.6
	No	109	7.4
Training duration	1–3 days	519	35.3
	4–7 days	457	31.0
	1–2 weeks	167	11.3
	More than 1 month	132	8.9
	2–4 weeks	86	5.8
Training hours	1–3 h	616	41.9
	More than 6 h	317	21.5
	4–6 h	235	15.9
	Less than 1 h	192	13.0
On-the-job training	Yes	1,279	87.0
	No	191	13.0
Training frequency	Once a month	843	57.3
	Once a quarter	208	14.1
	Once every 6 months	129	8.7
	Once a year	46	3.1
	Other	33	2.2
	Over 1 year	20	1.3
Centralized training method	Mentorship by senior nursing assistants	978	66.5
	Classroom lectures	932	63.4
	Knowledge and skills assessment	680	46.3
	Site visits	458	31.2
	Skill competitions	265	18.0
	WeChat groups/official accounts	258	17.6
	Mobile apps	200	13.6
	Computer platforms	139	9.5

### 3.2 Univariate analysis of training and retraining requirements

Univariate analysis (Table 2) showed that retraining needs were significantly associated with most demographic and work-related factors (all *p* < 0.05), except educational level. Lower retraining needs were reported by nursing assistants aged ≥ 70 years and those with more than 5 years of experience. In contrast, higher retraining needs were observed among participants holding professional certificates, working under one-on-one or one-to-many care models, and those employed by hospitals or third-party agencies. Notably, individuals who had received pre-employment or on-the-job training, or who were mentored by experienced nursing assistants, reported higher retraining needs, suggesting increased awareness of skill gaps following prior training exposure.

**TABLE 2:** Univariate analysis of factors associated with training and retraining requirements among nursing assistants.

Category	Content			Analysis	Result
		Requirement for training/retraining		χ^2^	*P*
		No requirement for training/retraining (*n* = 317)	Requirement for training/retraining (*n* = 1,153)		
Gender	Female	257	952	0.380	0.537
	Male	60	201		
Age	< 60	156	563	9.452	0.009
	60–69	115	486		
	> 70	46	104		
Educational level	Illiterate	53	222	4.602	0.203
	Primary school	138	482		
	Junior high school	91	284		
	High school or above	35	165		
Years of experience	< 1 year	61	278	23.459	< 0.001
	1–3 years	78	325		
	4–5 years	50	244		
	> 5 years	128	306		
Relevant certificates	No	111	304	9.182	0.002
	Yes	206	849		
Employment type	Hospital-employed	55	242	22.281	< 0.001
	Labor dispatch	237	883		
	Family-employed	25	28		
Work model	Team-based model	65	163	7.819	0.020
	One-on-one care	168	648		
	One-to-many care	84	342		
Pre-employment training	No	34	75	6.452	0.011
	Yes	283	1,078		
On-the-job training	No	83	104	66.068	< 0.001
	Yes	233	1,046		
Mentorship by other nursing assistants	No	80	132	45.731	< 0.001
	Yes	207	983		

### 3.3 Multivariate analysis of training and retraining requirements

**FIGURE 1:**
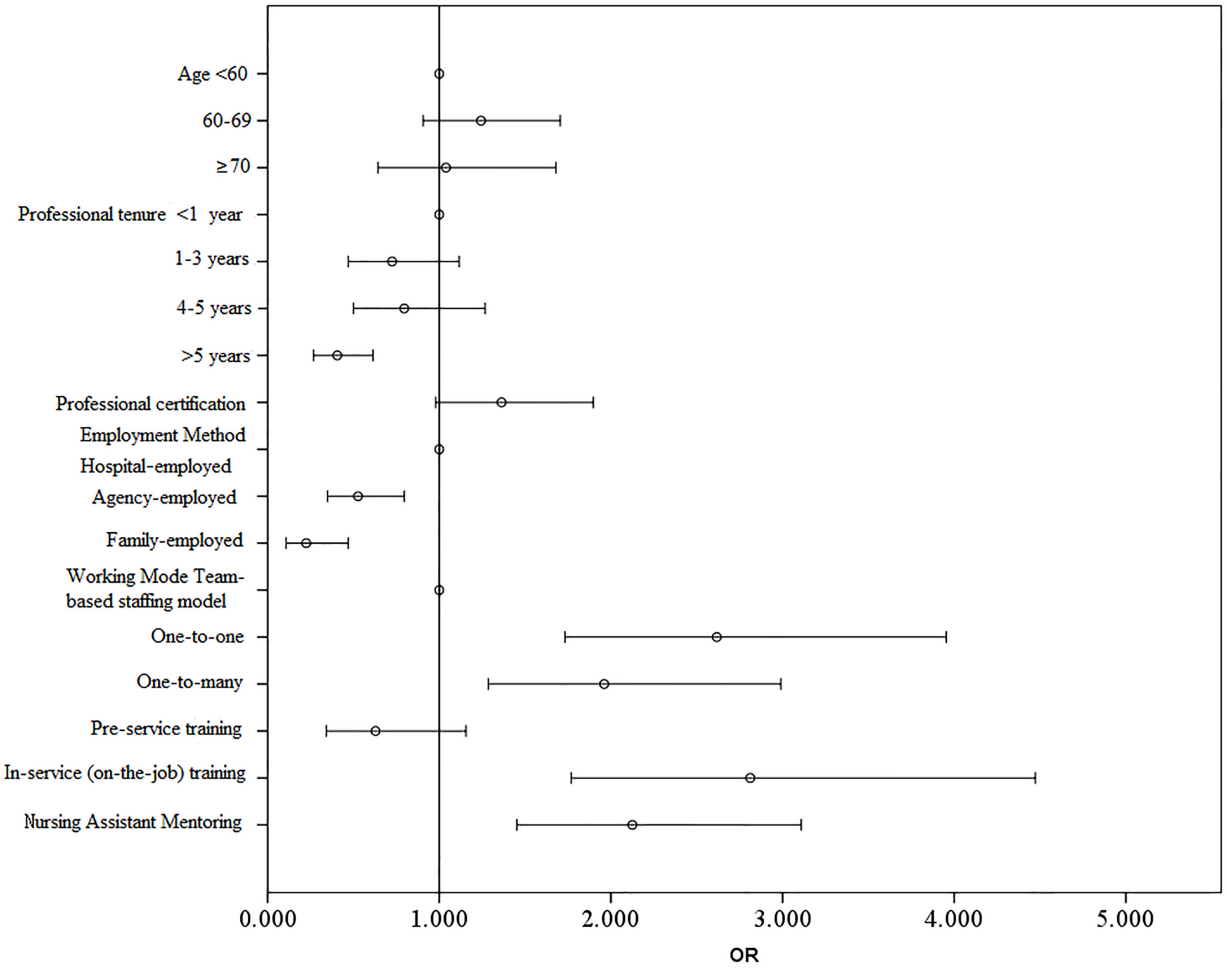
Adjusted odds ratios for factors influencing nursing assistants’ retraining intent (multivariate logistic regression). The forest plot shows adjusted odds ratios from multivariate logistic regression for factors associated with nursing assistants’ willingness to participate in retraining. Higher retraining intent was observed among assistants working in one-to-one or one-to-many care models, those who had completed on-the-job training, and those mentored by experienced caregivers (OR ≈ 2–4). Conversely, assistants with ≥ 5 years of experience or employed through agencies or directly by families showed lower retraining intent (OR < 1).

Variables significant in univariate analysis were entered into a multivariate logistic regression model (Table 3 and Figure 1). Longer work experience (> 5 years) and employment via labor dispatch or direct family hire remained independently associated with lower retraining needs. Conversely, one-on-one or one-to-many care models, prior on-the-job training, and mentorship by other nursing assistants were independently associated with higher retraining needs (all *p* < 0.05). These findings indicate that both work organization and prior training exposure play key roles in shaping perceived retraining demand.

**TABLE 3:** Multivariate logistic regression analysis of factors influencing retraining intent among nursing assistants.

Category	*B*	S.E.	Wald	*P*	OR	95% CI	
**Age**							
< 60					1.000		
60–69	0.217	16.10%	1.817	0.178	1.243	0.906	1.705
> 70	0.038	24.50%	0.024	0.876	1.039	0.642	1.68
**Years of experience**							
< 1 year					1.000		
1–3 years	−0.322	22.10%	2.124	0.145	0.725	0.47	1.117
4–5 years	−0.228	23.70%	0.922	0.337	0.796	0.5	1.268
> 5 years	−0.902	21.10%	18.214	< 0.001	0.406	0.268	0.614
Certificates	0.31	16.80%	3.385	0.066	1.363	0.98	1.897
**Employment type**							
Hospital-employed			1.000				
Labor dispatching	−0.64	21.00%	9.29	0.002	0.527	0.35	0.796
Family-employed	−1.491	37.60%	15.741	< 0.001	0.225	0.108	0.47
**Work model**							
Team-based model			1.000				
One-on-one care	0.962	21.10%	20.863	< 0.001	2.617	1.732	3.954
One-to-many care	0.673	21.50%	9.772	0.002	1.961	1.286	2.991
Pre-employment training	−0.464	31.00%	2.237	0.135	0.629	0.342	1.155
On-the-job training	1.034	23.70%	19.069	< 0.001	2.812	1.768	4.473
Mentorship by other nursing assistants	0.754	19.40%	15.044	< 0.001	2.125	1.452	3.110

### 3.4 Preferences of nursing assistants for retraining

Preferences for retraining are summarized in Table 4. Participants favored short-term programs (1–3 days; 49.5%) delivered on a monthly basis (56.3%). Senior nursing assistants (67.8%) and nursing experts (61.6%) were the most preferred trainers. Hospital training centers (64.6%) and inpatient wards (58.0%) were the preferred venues. Hands-on training, particularly bedside mentorship, was overwhelmingly favored (92.1%), whereas digital training tools were rarely used (< 20%). The most requested content areas were professional skills (67.3%), communication skills (59.2%), and patient safety (54.4%). Demand for specialized topics such as end-of-life care was comparatively lower (31.2%).

**TABLE 4:** Preferred training duration, frequency, format, venue, and content among nursing assistants in Jiaxing (*n* = 1,470).

Item	Options	Number of cases	(%)
Training format	Demonstrations and hands-on practice	1,174	79.9
	Multimedia lectures	699	47.6
	Scenario simulations (countermeasure simulations)	580	39.5
	Experiential learning	488	33.2
	Simulation-based training systems	468	31.8
	Role-playing	418	28.4
Training content	Qualities and requirements of nursing assistants	989	67.3
	Communication skills	870	59.2
	Job responsibility	859	58.4
	Basic protection	813	55.3
	Identification and prevention of patient safety risks	799	54.4
	Relevant laws, regulations, and policies	770	52.4
	Recognition of common patient symptoms	751	51.1
	Work-related emotional management	710	48.3
	Hand hygiene	699	47.6
	Medical waste disposal	690	46.9
	Use of wheelchairs	670	45.6
	Work task/time management	665	45.2
	Management of abnormal urination and defecation	633	43.1
	Body hygiene	630	42.9
	Cleaning and disinfection	628	42.7
	Bed unit arrangement	626	42.6
	Feeding care	626	42.6
	Care for patients with cognitive impairment	618	42.0
	Oral hygiene	615	41.8
	Pain management for patients	614	41.8
	Patient mobility	604	41.1
	Head hygiene	599	40.7
	Assistance with dressing	586	39.9
	Toileting care	578	39.3
	Use of positioning cushions	567	38.6
	Medication management	556	37.8
	Use of walkers	541	36.8
	Use of stretchers	539	36.7
	Use of ice packs and hot water bags	485	33.0
	End-of-life care	458	31.2
	Postmortem care	277	18.8

## 4 Discussion

The findings indicated high coverage rates of both pre-employment and on-the-job training among nursing assistants in Jiaxing. However, The length and depth of training were uneven. Most participants had completed pre-employment or on-the-job training, as reported by 92.6 and 87.2%, respectively, indicating that healthcare institutions in the region place considerable emphasis on professional training. Nevertheless, training duration was predominantly short, with 31.0% of participants receiving training lasting 4–7 days and 35.3% receiving training lasting only 1–3 days. This situation contrasts with the more systematic and structured training models established in developed countries such as the United States, the United Kingdom, Germany, and Japan (12–14). For example, the Health Education England Health Assistant Certification Program requires trainees to complete 15 competency-based modules within 12 weeks of employment, covering topics such as basic life support and infection control. These modules include both theoretical and practical components and culminate in certification. In addition, trainees must complete 10 online learning modules and practical sessions (15). In Germany, nursing care personnel are required to hold at least a secondary vocational school diploma, be 16 years or older, and be physically and mentally fit. Training must include a minimum of 2,100 h of theoretical instruction and 2,500 h of practical experience over at least 18 months, with alternating theoretical and clinical components (16). The limited duration and fragmented nature of current programs have led to suboptimal training outcomes, consistent with the findings of Li et al. (7).

Demographic characteristics among nursing assistants appeared to constrain the implementation of diversified training approaches. More than half of the participants were aged over 60 years (51.1%), with a majority being women. These characteristics contributed to the continued reliance on traditional training formats such as hospital-based lectures (63.4%) and mentorship provided by senior nursing assistants (66.5%). Moreover, limited use of digital instructional tools, including mobile applications and WeChat groups, hindered improvements in training accessibility and effectiveness, in line with previous observations (17).

Multiple factors were associated with training and retraining requirements. Univariate analysis demonstrated that nursing assistants aged 60–69 reported higher retraining requirements compared to those aged over 70. Similarly, those with shorter employment durations exhibited greater training demand than those with longer tenures. Individuals holding professional certificates expressed higher retraining requirements than their uncertified counterparts. Nursing assistants employed by hospitals or third-party agencies reported greater training requirements than those hired directly by families. The study found that practitioners working under one-to-one or one-to-many care models exhibited higher training needs than those engaged in team-based collaboration. Additionally, prior participation in pre-employment or on-the-job training and exposure to mentorship from experienced caregivers were associated with greater interest in retraining.

Multivariate logistic regression analysis confirmed that higher retraining requirements were significantly associated with caregivers working in one-on-one or one-to-many models, those who had previously received on-the-job training, and those mentored by peer caregivers (*p* < 0.05). These findings emphasize the influence of individual characteristics, workplace structures, and prior training experiences on ongoing educational needs.

### 4.1 Current preferred training models among nursing assistants in Jiaxing

#### 4.1.1 Training duration and frequency

Participants demonstrated a preference for short-term training lasting 1–3 days (49.5%), likely reflecting the high clinical workload and the need to acquire skills efficiently. Additionally, 56.3% preferred training to occur monthly, indicating a need for continuous skill updates in response to evolving clinical demands. These findings indicate that training programs should be designed to optimize the balance between instructional depth and time efficiency, minimizing disruption to routine clinical responsibilities.

#### 4.1.2 Training instructors

Senior nursing assistants (67.8%) and nursing experts (61.6%) were most frequently identified as preferred trainers. This reflects a dual emphasis on both practical experience and theoretical expertise. Practical instruction from experienced nursing assistants can promote procedural accuracy, while expert-led sessions can provide theoretical grounding. The integration of these two formats may form an iterative “practice-theory” cycle that enhances training effectiveness.

#### 4.1.3 Training venues and formats

Preferred venues included hospital training centers (64.6%) and inpatient wards (58.0%), indicating a preference for learning environments embedded in real clinical contexts. These venues facilitate direct observation and application of clinical workflows, thereby expediting the transfer of knowledge. Traditional bedside mentorship was the most favored instructional format (72.2%). However, the adoption of digital training tools remained limited (below 20%), indicating an area for improvement. Blended models incorporating virtual simulations alongside in-person mentorship could offer a more flexible and scalable training approach.

#### 4.1.4 Training content

Core areas of interest included professional skills and competencies (67.3%), communication techniques (59.2%), and patient safety practices (54.4%). These priorities align with essential role competencies for nursing assistants. In contrast, demand for training in specialized scenarios, such as end-of-life care, was relatively low (31.2%), possibly reflecting limited exposure to such situations in routine care. Nonetheless, the low demand highlights a potential training gap. Gradual introduction of advanced content, such as case-based modules for specialized scenarios, may improve preparedness and expand the scope of care delivery.

### 4.2 Comparison with existing literature on training needs of nursing assistants

Our findings align with prior studies showing that high training coverage does not necessarily translate into adequate competency development among nursing assistants. Although more than 90% of participants in Jiaxing reported receiving pre-service or in-service training, the programs were generally brief and heterogeneous, with most lasting fewer than 7 days. This contrasts sharply with the multi-week, standardized training curricula required in countries such as the United States, the United Kingdom, Germany, and Japan, where longer training durations allow for repeated practice, supervision, and skill consolidation (18–20). Consistent with Li et al., fragmented and short-term training models are associated with limited learning gains and substantial variability in care competence (21).

The demographic profile of the workforce further shapes training delivery and uptake. In line with previous reports, our cohort was predominantly older, with more than half aged ≥ 60 years, which partly explains the continued reliance on traditional teaching formats such as hospital lectures and senior-assistant mentoring. Digital training modalities, including mobile applications and social media platforms, were used by fewer than 20% of respondents. Similar patterns have been reported in other studies of aging care workforces, where limited digital literacy and the absence of age-adapted online content restrict the effectiveness of e-learning approaches (22).

Our analysis also adds nuance to the literature by identifying subgroups with distinct retraining needs. Nursing assistants aged 60–69 years, those with shorter work experience, professional certification, and hospital- or agency-based employment demonstrated stronger demand for retraining, whereas longer tenure, labor-dispatch arrangements, or direct family employment were associated with lower demand, even after adjustment for confounders (23). These findings are consistent with studies suggesting that job stability, perceived career progression, and organizational support strongly influence motivation for continued learning. Moreover, care models involving one-to-one or one-to-many assignments, prior in-service training, and active mentorship were associated with higher retraining interest, underscoring the role of workplace structure and supervisory support in sustaining learning motivation (24).

Finally, respondents’ preferences for retraining are largely consistent with international evidence emphasizing practicality and feasibility. Short, high-frequency, on-site training sessions led by experienced nursing assistants or clinical experts were favored, with strong emphasis on hands-on practice. Core competencies, communication skills, and patient safety were prioritized over specialized topics such as end-of-life care, echoing findings from similar workforce surveys. While digital tools remain underutilized, the literature suggests that blended approaches, combining face-to-face instruction with simple, case-based digital resources, may gradually expand training reach and address unmet educational needs without increasing cognitive or technological burden (25).

### 4.3 Limitations

Several limitations should be noted. First, although this study provides a large, city-wide snapshot of training utilization and retraining demand among nursing assistants, its cross-sectional design precludes causal inference. Second, data were collected using a self-administered questionnaire with a relatively large number of items, which may have increased respondent burden, the risk of input errors, and susceptibility to recall or social-desirability bias. Third, while stratified sampling was conducted across 48 healthcare institutions in Jiaxing, an economically developed city in China’s Yangtze River Delta region, the findings may not be fully representative of nursing assistants in less developed or rural areas. Differences in workforce structure, training resources, and institutional support may limit the generalizability of the results. Future studies using longitudinal designs, streamlined instruments, and multi-regional samples are needed to strengthen causal interpretation and external validity.

## 5 Conclusion

This study shows that while the overall coverage of nursing assistant training in Jiaxing is high, important gaps remain in training duration, delivery methods, content relevance, and sustained participation. These limitations reduce the practical impact of current programs despite their broad reach. Given the study’s cross-sectional design and reliance on self-reported data, the findings should be interpreted as descriptive rather than causal. From a practical perspective, training policies should shift from an emphasis on coverage to a focus on quality. Extending course length, standardizing core curricula, and integrating low-threshold digital resources may improve training effectiveness. A tiered strategy could prioritize new entrants, certificate holders, and nursing assistants in personalized care roles as early adopters, while experienced or family-employed assistants may require tailored incentives to re-engage. Embedding mentorship and short, regular training sessions within routine ward workflows offers a feasible approach to sustainable skill development. Future research should employ longitudinal or interventional designs and include diverse regions to assess training effectiveness over time and improve generalizability. Drawing on international long-term care workforce models, the development of a structured, standardized training and assessment framework aligned with China’s healthcare context may support the professionalization and long-term sustainability of the nursing assistant workforce.

## Data Availability

The original contributions presented in the study are included in the article/Supplementary material, further inquiries can be directed to the corresponding author.
