# Supplementary material for: Training requirements and associated factors for nursing assistants in Jiaxing City, China: a cross-sectional study in healthcare settings
Source: Front Med (Lausanne). 2026 Feb 12;13:1710478. doi: 10.3389/fmed.2026.1710478 (PMC12935655; doi:10.3389/fmed.2026.1710478)
Supplement: Supplementary file 1 [file Table_1.doc]

**In-Service Training Status Questionnaire for Nursing Assistants**

**Variable domains included:** demographic variables, occupational background variables, training experience variables, and work pattern variables.

**I. Demographic and background variables**

1. Sex (categorical variable: male, female)

2. Age (continuous variable / open-ended)

3. Place of origin (nominal variable / open-ended)

4. Educational level (ordinal categorical variable: never attended school → junior college or above)

5. Years of employment (ordinal categorical variable: less than one month → more than ten years)

6. Previous occupation (multiple-choice categorical variable: farming, domestic helper, self-employed, factory worker, etc.)

**II. Occupational and certification variables**

1. Service specialty/department (categorical variable: orthopedics, hepatobiliary surgery, etc.)

2. Type of certificate held (multiple-choice categorical variable: none, employment permit, nursing assistant certificate, etc.)

3. Certificate level (ordinal categorical variable: junior, intermediate, senior)

4. Existence of a nursing assistant grading system in the hospital (categorical variable: yes, no, unclear)

5. Details of the nursing assistant grading system (open-ended / text variable)

6. Employment mode (categorical variable: hospital-employed, third-party agency, patient/family-employed)

7. Work model (categorical variable: one-to-one care, one-to-many care, team-based care)

**III. Training experience variables**

1. Received school-based education or training (binary: yes/no)

2. Received pre-employment training (binary: yes/no)

3. Duration of pre-employment training (ordinal categorical variable: 1–3 days → more than one month)

4. Daily length of pre-employment training (ordinal categorical variable: less than 1 hour → more than 6 hours)

5. Location of pre-employment training (multiple-choice categorical variable: school, hospital, nursing home, etc.)

6. Instructors for pre-employment training (multiple-choice categorical variable: university teachers, hospital nurses, company trainers, etc.)

7. Received in-service training (binary: yes/no)

8. Frequency of in-service training (ordinal categorical variable: once per month → less than once per year)

9. Location of in-service training (multiple-choice categorical variable: school, hospital, company, etc.)

10. Instructors for in-service training (multiple-choice categorical variable: same as above)

11. Difficulties encountered during training (multiple-choice categorical variable: unengaging content, inconvenient timing, lack of support, etc.)

12. Received mentorship from experienced nursing assistants (binary: yes/no)

13. Training modalities participated in (multiple-choice categorical variable: classroom teaching, mentorship, WeChat groups, apps, etc.)

14. Formats of centralized teaching (multiple-choice categorical variable: multimedia lectures, skills demonstrations, scenario simulations, etc.)

15. Preferred work schedule (categorical variable: 24-hour continuous care, two-shift system, three-shift system)

**In-Service Training Needs Questionnaire for Nursing Assistants**

**Variable domains included:** training willingness variables, training preference variables, and content demand variables.

**I. Training willingness and barriers**

1. Need for training (binary: yes/no)

2. Reasons for no training need (multiple-choice categorical variable: content already mastered, lack of time, lack of managerial support, etc.)

**II. Preferences for training structure and format**

1. Acceptable duration of pre-employment training (ordinal categorical variable: 1–3 days → more than one month)

2. Preferred frequency of in-service training (ordinal categorical variable: once per month → once per year)

3. Preferred trainer identity (multiple-choice categorical variable: nursing experts, senior nursing assistants, university faculty, etc.)

4. Preferred training venue (multiple-choice categorical variable: hospital ward, hospital training center, school, etc.)

5. Preferred training methods (multiple-choice categorical variable: classroom teaching, on-site mentorship, online platforms, etc.)

6. Preferred formats for centralized teaching (multiple-choice categorical variable: multimedia lectures, skills demonstrations, scenario simulations, etc.)

**III. Training content needs**

1. Required training topics (multiple-choice categorical variable: laws and regulations, communication skills, hand hygiene, patient transfer, etc.; a total of 30 specific items)

2. Suggestions for training (open-ended / text variable)
